# Inflammatory CD4/CD8 double-positive human T cells arise from reactive CD8 T cells and are sufficient to mediate GVHD pathology

**DOI:** 10.1126/sciadv.adf0567

**Published:** 2023-03-24

**Authors:** Nicholas J. Hess, David P. Turicek, Jeremiah Riendeau, Sean J. McIlwain, Emmanuel Contreras Guzman, Kalyan Nadiminti, Amy Hudson, Natalie S. Callander, Melissa C. Skala, Jenny E. Gumperz, Peiman Hematti, Christian M. Capitini

**Affiliations:** ^1^ Department of Pediatrics, University of Wisconsin School of Medicine and Public Health, Madison, WI, USA.; ^2^ Department of Medicine, University of Wisconsin School of Medicine and Public Health, Madison, WI, USA.; ^3^ University of Wisconsin Carbone Cancer Center, Madison, WI, USA.; ^4^ Morgridge Institute for Research, Madison, WI, USA.; ^5^ Department of Biomedical Engineering, University of Wisconsin-Madison, Madison, WI, USA.; ^6^ Department of Biostatistics and Medical Informatics, University of Wisconsin-Madison, Madison, WI, USA.; ^7^ Department of Microbiology and Immunology, Medical College of Wisconsin, Milwaukee, WI, USA.; ^8^ Department of Medical Microbiology and Immunology, University of Wisconsin School of Medicine and Public Health, Madison, WI, USA.

## Abstract

An important paradigm in allogeneic hematopoietic cell transplantations (allo-HCTs) is the prevention of graft-versus-host disease (GVHD) while preserving the graft-versus-leukemia (GVL) activity of donor T cells. From an observational clinical study of adult allo-HCT recipients, we identified a CD4^+^/CD8^+^ double-positive T cell (DPT) population, not present in starting grafts, whose presence was predictive of ≥ grade 2 GVHD. Using an established xenogeneic transplant model, we reveal that the DPT population develops from antigen-stimulated CD8 T cells, which become transcriptionally, metabolically, and phenotypically distinct from single-positive CD4 and CD8 T cells. Isolated DPTs were sufficient to mediate xeno-GVHD pathology when retransplanted into naïve mice but provided no survival benefit when mice were challenged with a human B-ALL cell line. Overall, this study reveals human DPTs as a T cell population directly involved with GVHD pathology.

## Graphical abstract

**Figure:**
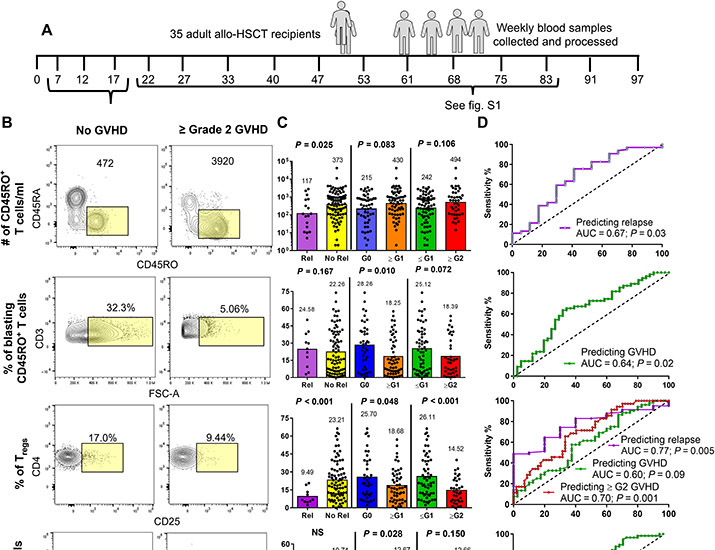

## Teaser

Human CD4+/CD8+ double positive T cells (DPTs) mediate xenogeneic GVHD but possess limited GVL activity.

## INTRODUCTION

Graft-versus-host disease (GVHD) and relapse remain the primary complications following allogeneic hematopoietic cell transplantation (allo-HCT) (*1*–*3*). While the field has made substantial strides in reducing GVHD, current GVHD prophylaxis drug regimens target the entire T cell population, potentially hindering the efficacy of graft-versus-leukemia (GVL) activity mediated by donor T cells (*4*–*8*). Thus, identifying predictive biomarkers and delineating specific cellular mechanism(s) leading to GVHD pathology versus GVL activity has remained a top priority for the field.

Unfortunately, separating the GVH response of donor T cells from their GVL activity has proven difficult as both responses are derived from allogeneic antigen stimulation (*7*–*9*). Nevertheless, several studies have investigated the role of specific human leukocyteantigen (HLA) mismatches on the development of GVHD and GVL activities (*10*–*13*). These studies have highlighted the importance of permissive and nonpermissive HLA mismatches on GVHD development and nonrelapse mortality, but correlating them with GVL activity has been met with limited success (*10*–*14*). Furthermore, the use of haploidentical transplantations with posttransplant cyclophosphamide has shown that HLA matching of the donor and recipient may not play a dominant role in separating GVH and GVL activity (*15*, *16*).

While pretransplant prognostic variables such as HLA matching, conditioning regimens, and graft source all influence allo-HCT outcomes, these population-based variables are not able to predict individual patient outcomes (*17*–*19*). Several posttransplant predictive variables have been found, including sST2, REG3α, elafin, and others (*20*–*26*). These highly promising plasma-based biomarkers are generally released because of host damage and have shown efficacy in predicting the development of steroid-resistant GVHD and nonrelapse mortality either individually or combined into a predictive algorithm (*20*–*26*). To date, though, these biomarkers have not been able to predict an allo-HCT recipients’ chance of relapse as they measure the degree of host damage rather than donor T cell alloreactivity.

While all the validated GVHD biomarkers to date have been plasma based, the direct investigation of T cell subsets as biomarkers has been another area of active investigation (*27*–*30*). Both CD4 and CD8 T cells can respond to allogeneic antigens, but while CD8 T cells may be directly responsible for cellular cytotoxicity, CD4 T cells have been the primary focus of the field (*31*–*33*). The CD4 population is unique in its capacity to polarize into a variety of different subsets, based on the cytokine environment it finds itself, to drive an inflammatory response (*34*–*40*). In the case of GVHD, T helper 1 (T_H_1) and T_H_17/22 cells have been shown to be directly involved in driving the pathogenesis of gastrointestinal, liver, and skin pathology (*35*, *41*, *42*). In contrast, the CD8 T cell population is thought to be more conserved in their role as cytotoxic effectors licensed by CD4 T cells. The discovery of CD8 regulatory T cells (T_regs_) and Tc17 cells in murine GVHD though suggests that CD8 T cells may also be able to polarize into different subsets similar to the CD4 population (*43*–*45*). T cell–specific GVHD prophylaxis regimens have been (e.g., anti-thymocyte globulin) and are currently (e.g., abatacept) promising therapeutics, but the next generation of T cell–targeting GVHD prophylaxis regimens will need to precisely target GVHD-specific T cell populations.

Unexpectedly, there have been numerous studies in the past 2 decades that have observed the presence of a T cell population coexpressing both the CD4 and CD8 lineage markers. Their presence has been identified in a range of human chronic inflammatory diseases including most recently in GVHD (*46*–*56*). While historically thought to be the result of thymocytes escaping the thymus, recent observational studies suggest that CD4^+^/CD8^+^ double-positive T cells (DPTs) are a mature human T cell population that develops in the periphery in response to antigenic stimulation (*57*, *58*).

In this study, we identified DPTs as a predictive biomarker of GVHD in a 35-patient observational study of allo-HCT recipients at a single institution. Using a xenogeneic transplant model system to directly investigate human T cell biology, we further investigated the functional relevance of DPTs in GVHD pathogenesis (*31*, *59*). We identify the origin of DPTs, which are not present in healthy human grafts, as the CD8 T cell population that arises in response to T cell receptor (TCR)–dependent activation signals. We also provide evidence that the transcriptional, metabolic, and phenotypically distinct DPTs are sufficient to mediate xeno-GVHD pathology but provide no survival advantage when mice are also challenged with a human B cell acute lymphoblastic leukemia (B-ALL) malignancy. Overall, this study provides the first comprehensive analysis of the origin, effector functions, and clinical significance of a human DPT population.

## RESULTS

### Increased frequencies of CD4^+^/CD8^+^ DPTs are predictive of GVHD in an observational clinical cohort of allo-HCT recipients

**Fig. 1.**
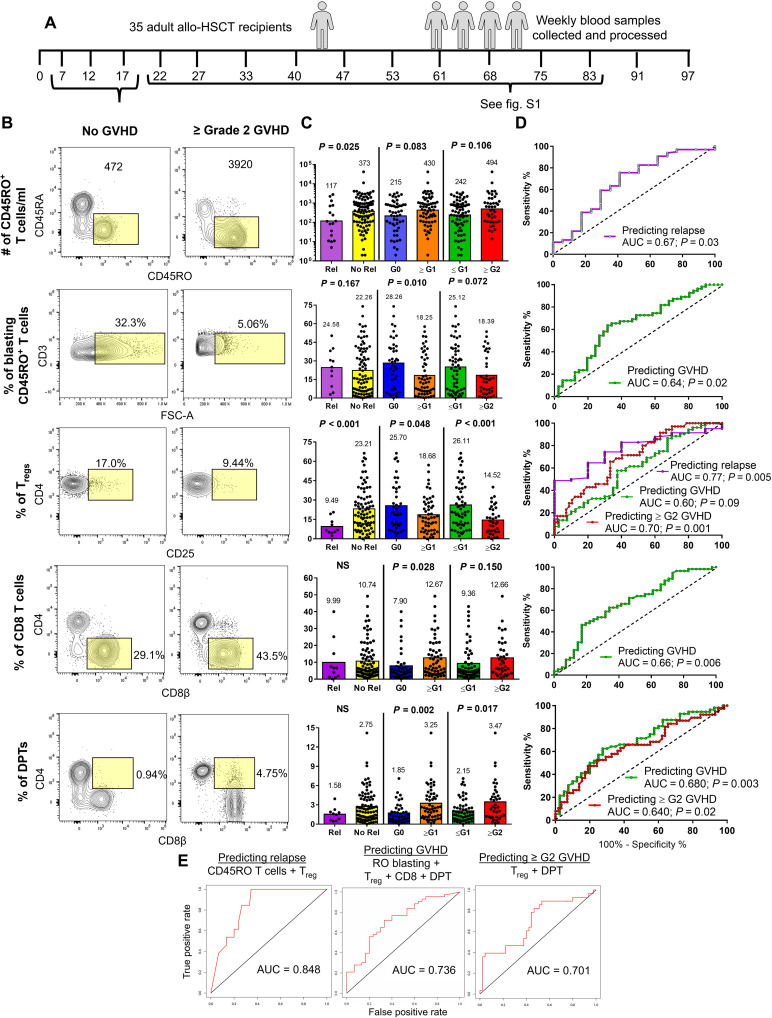
Posttransplant T cell metrics are predictive of allo-HCT recipient disease outcomes. (**A**) Schematic depicting the study outline. Thirty-five allo-HCT recipients had weekly blood samples collected and processed without freezing. (**B**) Dot plots taken from five concatenated allo-HCT recipient samples that did not develop GVHD and those that developed ≥ grade 2 GVHD. Yellow box indicates gating strategy for each T cell metric. Samples collected between the day 7 and 17 collection intervals were used for the analysis in (**C** to **E**), while the analysis for samples collected between days 22 and 83 can be found in fig. S3. (C) Each T cell metric was analyzed in three binary tests comparing two allo-HCT outcomes. Mean and significance value are shown above each test, with each data point representing an individual sample. (D) Tests reaching a significance of *P* < 0.05 were further analyzed by ROC, with the AUC and significance value shown. (E) T cell metrics predicting the same allo-HCT outcome were combined into a multiparameter ROC analysis, with the test, metrics used, and AUC values shown in the graphs. ROC, receiver-operator characteristic; AUC, area under the curve; G0-G2 indicates highest grade of GVHD achieved by the patient; NS, not significant.

To determine whether the direct measurement of T cells in allo-HCT recipients after transplant can serve as predictive biomarkers of relapse and/or GVHD, we collected 417 blood samples from 35 adult allo-HCT recipients over their first 100 days after transplant (Fig. 1A). Patients were included in the observational study irrespective of their conditioning regimen, donor HLA matching, graft source, or GVHD prophylaxis regimen. The cohort is heavily skewed to patients receiving granulocyte colony-stimulating factor (G-CSF) mobilized peripheral blood and a cyclophosphamide-based GVHD prophylaxis regimen as both conditions are standard at our clinic (table S1). An average of 12 samples were collected per patient and a total of 25 samples per collection interval (~every 4 to 6 days) (fig. S1). Additional information on the stratification of this cohort can be found in Materials and Methods. Samples were processed within 7 days of collection and never frozen. Antigen-experienced T cells were gated on the basis of CD3 and CD45RO expression to exclude nonreactive donor T cells and naïve T cells that are present because of de novo hematopoiesis (fig. S2).

From a total of 11 T cell metrics analyzed, we identified five metrics as having the capacity to predict relapse, GVHD (of any grade), or ≥ grade 2 GVHD. These included the number of CD45RO^+^ T cells and the frequencies of blasting CD45RO^+^ T cells, T_regs_, CD8^+^ T cells, and DPTs (Fig. 1B and fig. S3). We first analyzed the samples collected between days 7 and 17 in three binary tests to determine whether any biomarkers could differentiate between two allo-HCT outcomes (Fig. 1C). Specific comparisons that were shown to be significant by *t* test were further evaluated using a receiver-operator characteristic (ROC) analysis (Fig. 1D). We identified two predictive biomarkers of relapse (CD45RO T cells and frequency of T_regs_), four predictive biomarkers of GVHD (CD45RO T cells and the frequency of T_regs_, CD8, and DPTs), and two predictive biomarkers of ≥ grade 2 GVHD (frequency of T_regs_ and DPTs) (Fig. 1, C and D). Combining the individual T cell metrics into one multiparameter analysis further increased their predictive potential as measured by the area-under-the-curve (AUC) values that reached 0.848, 0.736, and 0.701 for relapse, GVHD, and ≥ grade 2 GVHD prediction, respectively (Fig. 1E).

A second analysis included patient samples collected between days 22 and 83 and were further normalized relative to each individual patient’s date of grade 1 or 2 GVHD diagnosis. For patients who did not develop GVHD, we normalized their data to day 54, which was the average time to GVHD diagnosis among our cohort. In this analysis, only three of the original five T cell biomarkers showed any predictive value with all three metrics predicting relapse (CD45RO T cells and the frequencies of CD8 and DPTs) and the increased frequency of CD8 and DPTs predicting both GVHD (of any grade) and ≥ grade 2 GVHD (fig. S3). Combining the predictive metrics into one multiparameter analysis as before further increased their predictive value with AUC values of 0.892, 0.741, and 0.734 for relapse, GVHD, and ≥ grade 2 GVHD prediction, respectively (fig. S3).

### Xenogeneic transplantation also reveals DPTs as a predictive biomarker of GVHD development

**Fig. 2.**
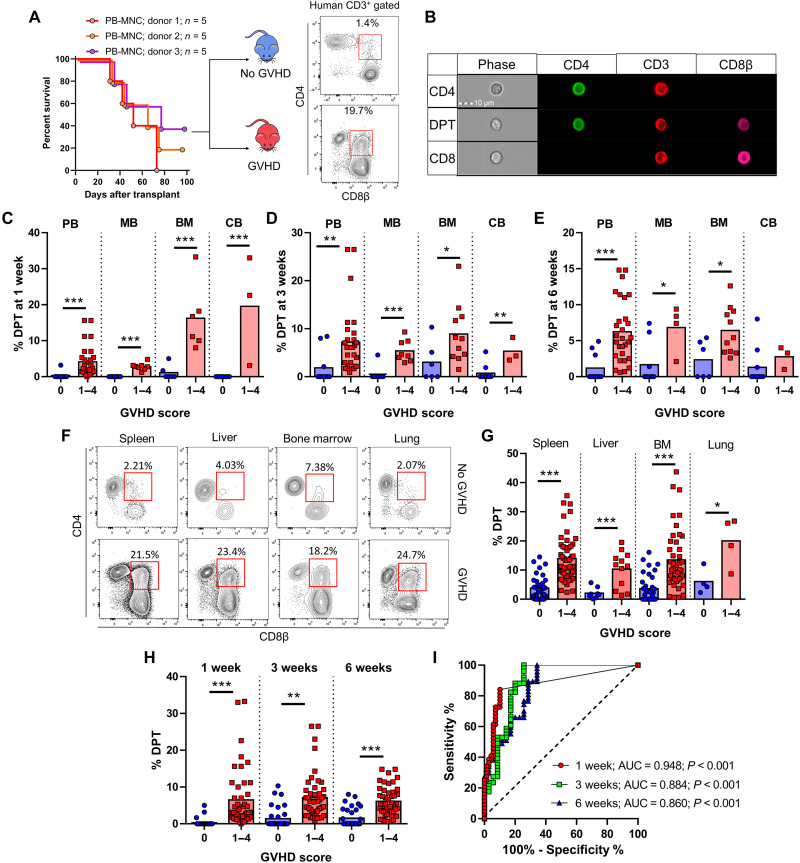
DPTs develop after xenogeneic transplantation and are predictive of lethal GVHD. (**A**) Survival curve of mice transplanted with PB-MNC from three different donors. Mice were monitored for signs of xeno-GVHD and retrospectively assigned into the no GVHD (blue) or xeno-GVHD (red) group based on their survival at 12 weeks after transplant. Representative dot plots are of human pan-HLA and CD3 gated mouse blood at 3 weeks after transplant, with the red box indicating the DPT gate. (**B**) ImageStream visualization of human T cells isolated from mouse blood. (**C** to **E**) Graphical representation of the percentage of DPTs in the blood of mice transplanted with the indicated graft source at 1 week (C), 3 weeks (D), and 6 weeks (E) after transplant. (**F** and **G**) Dot plots and graph of the percentage of DPTs in the indicated organs of mice at the time of euthanasia. Red squares indicate DPT gate. (**H** and **I**) Blood data from (C) to (E) were combined (H) and analyzed by ROC (I), with the AUC and significance values indicated. Each point represents an individual mouse. PB (peripheral blood), MB (G-CSF mobilized peripheral blood), BM (bone marrow), CB (umbilical cord blood), MNC (mononuclear cells). Parametric *t* test was used to determine significance. *P < 0.05, **P < 0.01, and ***P < 0.001.

To replicate our clinical observational study, we used a human xenogeneic transplant model wherein we transplanted various human graft tissues into immunodeficient mice (*31*, *59*, *60*). As we have published previously, xenogeneic GVHD (xeno-GVHD) is dose dependent with each human graft source having its own median lethal dose (LD_50_) and that the development of xeno-GVHD is not donor dependent (*59*). Sampling the blood of mice transplanted with human peripheral blood mononuclear cells (PB-MNC) at regular intervals, we divided mice into those that developed lethal xeno-GVHD and those that did not based on their survival at 12 weeks after transplant (Fig. 2A). We observed the presence of DPTs, which were not present in the starting healthy graft tissue, primarily developing in mice that would later develop lethal xeno-GVHD (Fig. 2A). We confirmed that the development of DPTs is not dependent on a single human donor, that DPTs are not artifacts of doublets by ImageStream analysis, and that DPTs develop even when isolated human T cells are used for transplantation (Fig. 2, A and B, and fig. S4). DPTs were present in a variety of xeno-GVHD target organs and developed as early as 1 week after transplant in each of the four graft sources we tested (Fig. 2, C to H). The increased frequency of DPTs was also a predictive biomarker of lethal xeno-GVHD development in this model system at 1, 3, and 6 weeks after transplant (Fig. 2I). We further confirmed that the frequency of T_regs_ 1 week after transplant could act as a predictive biomarker of lethal xeno-GVHD, but with the contribution of T_regs_ already well established in the GVHD literature, we chose to focus on the origin and function of DPTs for the remainder of this study (fig. S5).

### The DPT population is transcriptionally distinct from CD4 and CD8 T cells

**Fig. 3.**
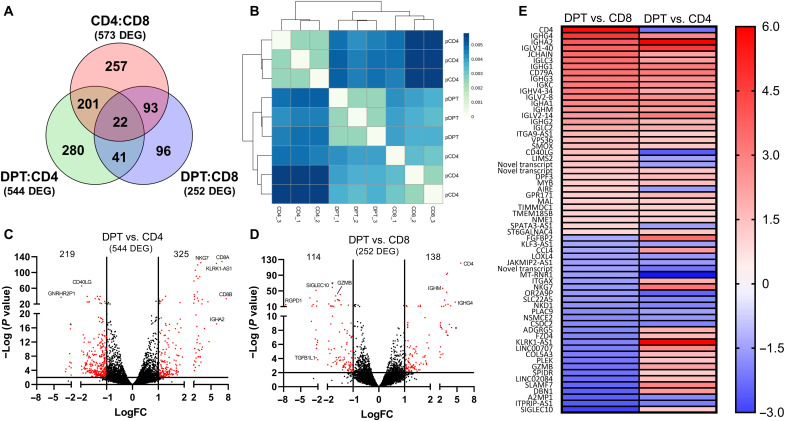
The transcriptome of DPT is unique relative to CD4 and CD8 T cells. (**A**) Venn diagram of the number of independent and shared differentially expressed genes among the three comparisons indicated. (**B**) Unsupervised hierarchical clustering of the RNA-seq samples using the computed Pearson correlation for each pair of samples and the Euclidean distances of the correlation distances determined. (**C** and **D**) Volcano plots showing the number of differentially expressed genes in the DPT:CD4 (C) and DPT:CD8 (D) comparisons using a cutoff of a ≥1 log expression difference and a *P* value of 0.01. (**E**) List of the 63 genes differentially expressed in both the DPT:CD4 and DPT:CD8 comparisons, with red indicating increased expression in DPTs relative to CD4/CD8 T cells and blue indicating decreased expression.

To explore whether the CD4 and CD8 lineage markers are acquired via trogocytosis or de novo transcribed, we performed RNA sequencing (RNA-seq) on flow-sorted CD4, CD8, and DPTs from xeno-GVHD mice with a sort purity of >98% (Fig. 3). Analysis of the transcriptome from these cells revealed that DPTs express a transcriptional signature unique from CD4 or CD8 T cells (Fig. 3, A and B). Using a threshold of *P* < 0.01 and a fold change of >10 to determine significance, we reveal that DPTs differentially expressed 63 genes when compared to both CD4 and CD8 T cells (Fig. 3A). Of the differentially expressed genes, we confirmed that both CD4 and CD8 are transcriptionally expressed in DPTs (Fig. 3, C to E). The longevity of CD4, CD8α, and CD8β coexpression on isolated DPTs was also maintained in culture for up to 3 weeks and for at least 9 weeks when retransplanted into naïve NSG mice. These data further suggest that coexpression of the CD4 and CD8 lineage markers is not an acquired feature but produced de novo (fig. S6).

**Fig. 4.**
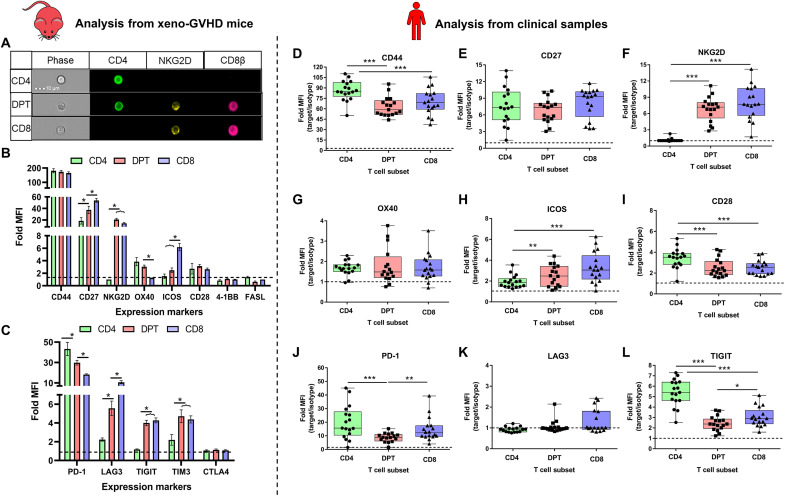
DPTs display a transitional profile with limited inhibitory receptor expression. (**A**) ImageStream visualization of DPTs expressing NKG2D. (**B** and **C**) Fold MFI (median fluorescent intensity of marker/isotype control) of CD4 (green), DPT (red), and CD8 T cell (blue) expression of eight proinflammatory (B) and five inhibitory (C) receptors analyzed from five to eight xeno-GVHD mice at 3 weeks after transplant. (**D** to **L**) Analysis of six proinflammatory (D to I) and three inhibitory receptors (J to L) on the surface of CD4 (green), DPT (red), or CD8 (blue) T cells from the allo-HCT patient samples collected as part of our observational study. Each dot represents the average Fold MFI of that marker across all samples collected for one individual patient. A minimum of 10 patients was collected for each marker. Expression of 4-1BB, FASL, and CTLA4 had no expression over an isotype control and is not shown. TIM3 was not measured in the clinical samples. Error bars represent SEM. Parametric *t* test was used to determine significance. *P < 0.05, **P < 0.01, and ***P < 0.001.

Using all 796 genes differentially expressed in the DPT:CD4 and DPT:CD8 comparisons, the database KEGG BRITE functional hierarchical classification highlighted most of the differentially expressed genes as having an enzymatic function, followed by CD molecules, exosome machinery, transcription factors, and cytokine/cytokine receptors (table S2). DPTs also appear to express several genes normally associated with B cell biology, although the mechanism for this observation requires further investigation (Fig. 3E). To confirm the differential expression of several inflammatory and inhibitory co-receptors, we surveyed 13 markers on the surface of CD4, CD8, and DPTs from xeno-GVHD mice (Fig. 4, A to C, and fig. S7). DPTs displayed an intermediate expression profile of CD27, OX40, PD-1, and LAG3 compared to CD4 and CD8 T cells analyzed from the blood or spleen of xeno-GVHD mice (Fig. 4, A to C, and fig. S6). Unexpectedly, DPTs did not express elevated levels of the inhibitory markers TIGIT, TIM3, or CTLA4 commonly expressed on exhausted T cells. The expression of these markers was further explored on the T cells from our observational clinical study (Fig. 4, D to L). While the expression of these markers differed slightly, most likely because of differences in murine versus human host environments, DPTs still displayed an expression profile of a terminally differentiated effector T cell population with little evidence of exhaustion (Fig. 4, D to L).

### Antigen-stimulated CD8 T cells develop into DPTs

**Fig. 5.**
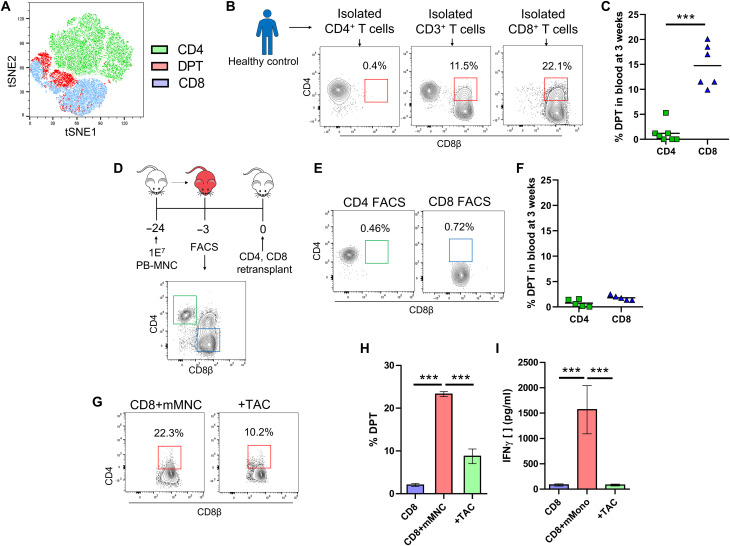
DPTs develop from CD8 T cells after antigenic stimulation. (**A**) Unsupervised tSNE clustering of human T cells concatenated from five xeno-GVHD mice. (**B** and **C**) Isolated CD3, CD4, and CD8 PB-Tc from a healthy donor were transplanted into mice. Dot plots show the percentage of DPTs in mouse blood at 3 weeks after transplant (B) with the percentage of DPT quantified in (C). (**D**) Five mice transplanted with PB-MNC were euthanized, and the human cells were isolated before flow sorting of CD4 and CD8 T cells. (**E** and **F**) Dot plots and bar graph of the percentage of DPTs in the blood of mice retransplanted with flow-sorted CD4 and CD8 T cells. (**G** to **I**) CD8 T cells were cocultured for 5 days with mouse MNC with or without tacrolimus and then analyzed for DPTs (H) and IFN-γ (I). Parametric *t* test was used to determine significance. ***P < 0.001.

To identify the T cell population that gives rise to DPTs, we performed an unsupervised t-distributed stochastic neighbor embedding (tSNE) clustering of human T cells from a xeno-GVHD mouse using a nine-color flow cytometry panel (Fig. 5A). This analysis, in addition to the global transcriptomic analysis, indicated that DPTs cluster more closely with CD8 T cells than CD4 T cells. Thus, despite the well-documented plasticity of the CD4 lineage, our results suggest that DPTs may develop from the CD8 lineage (Figs. 3B and 5A).

To test this hypothesis, we transplanted isolated CD4 or CD8 T cells from a healthy donor into our xenogeneic transplant model. Elevated frequencies of DPTs were only observed in mice transplanted with isolated CD8 T cells, indicating that mature CD8 T cells and not CD4 or progenitor T cells give rise to the DPT population (Fig. 5, B and C). To investigate whether the development of DPTs is antigen dependent or the result of a lymphopenic/xenogeneic environment, we first surveyed the phosphorylation status of several signal transducer and activator of transcription (STAT) proteins downstream of cytokine receptors commonly expressed by T cells. Unfortunately, we could not identify any differences in the phosphorylation of STAT1, STAT3, STAT4, and STAT6 between the CD8 and DPT populations. While the xenogeneic environment may still be playing a role in cytokine receptor/STAT activation, these data suggest that there is not any differential activation between the CD8 and DPT populations (fig. S8, A and B). To test the role of the xenogeneic environment further, we transplanted CD4 or CD8 T cells sorted from a xeno-GVHD mouse into a naïve mouse to determine whether DPTs would reform after the secondary transplant. We could not detect any development of DPTs in the mice receiving sorted CD8 T cells (Fig. 5, D to F). We next cocultured CD8 T cells in the presence of mouse MNCs for 5 days with or without tacrolimus to inhibit TCR signaling. The data revealed that mouse MNCs in culture could induce both DPT development and interferon-γ (IFN-γ) secretion, which was inhibited by tacrolimus (Fig. 5, G to I). These data suggest that DPTs are developing as a result of antigenic/xenogeneic stimulation by the TCR and not as a direct result of a lymphopenic/xenogeneic environment.

Knowing that the transcriptome of DPTs is unique compared to CD8 T cells and that transcription factors were one of the functional classes highlighted in the KEGG BRITE analysis, we further investigated what transcription factors may be involved in the differentiation of DPT from the CD8 population. Unfortunately, we were not able to definitively identify a transcription factor that was directly correlated with the development of DPTs (fig. S8, C to G). Relative to CD4 T cells, the DPT population had higher transcriptional expression of RUNX3 and T-bet and lower expression of FOXP3. Compared to CD8 T cells, only THPOK was statistically different, with DPTs having a higher level of THPOK transcripts (fig. S8C). To confirm these findings, we performed intracellular flow cytometry and Western blotting on a subset of the transcriptional factors identified by the RNA-seq data. The protein level expression differences of RUNX3 were confirmed but were inconclusive regarding THPOK due to the overall low expression level of the transcription factor (fig. S8, E to G). Thus, while the transcriptional expression of THPOK in DPTs is interesting, additional studies will be needed to determine the importance of THPOK in DPT development.

### Differentiated DPTs are a metabolically active and unique T cell population

**Fig. 6.**
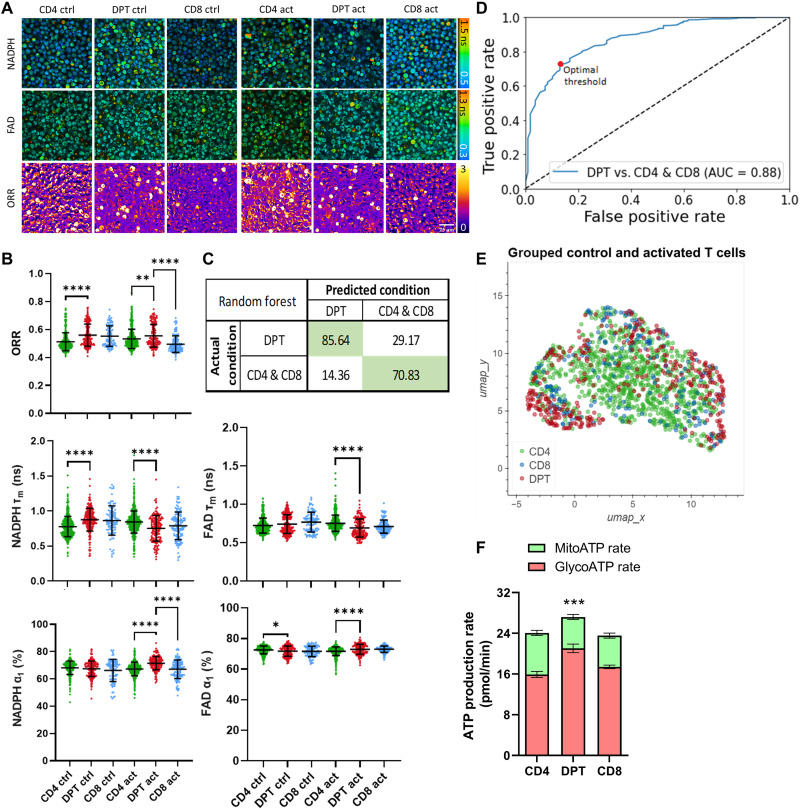
DPT cells exhibit a metabolically distinct signature from CD4 and CD8 T cells. (**A**) Representative images from live-cell imaging of NADPH mean lifetime (τ_m_, 0.5 to 1.5 ns), FAD τ_m_ (0.3 to 1.3 ns), and ORR images from flow-sorted CD4, CD8, and DPT from xeno-GVHD mice. All cells were cultured for 48 hours before visualization, with activated cells also stimulated with CD3/CD28/CD2 12 hours before imaging. Scale bar, top left, 25 μm. (**B**) Scatterplots of the ORR (NADPH/NADPH + FAD), NADPH τ_m_, FAD τ_m_, % unbound NADPH, and % protein-bound FAD. Each dot represents a single cell with mean at center and error bars representing 1 SD. Confusion matrix (**C**) and ROC curve (**D**) of a random forest classifier trained on the 10 optical metabolic imaging (OMI) parameters (NADPH and FAD τ_1_, τ_2_, α_1_, α_2_, and τ_m_). (**E**) UMAP dimensionality reduction analysis of OMI parameter results (τ_1_, τ_2_, α_1_, α_2_, and τ_m_) for both NADPH and FAD. (**F**) Seahorse analysis of the ATP production rate of flow-sorted CD4, DPT, and CD8 T cells. Parametric *t* test was used to determine significance. *P < 0.05, **P < 0.01, ***P < 0.001, and ****P < 0.0001. Cell counts ranged from 103 to 598 for each population.

To further explore DPT biology, we investigated their metabolic state relative to CD4 and CD8 T cells. We analyzed and sorted CD4, CD8, and DPTs from xeno-GVHD mice using a two-photon autofluorescence imaging system (Fig. 6, A to E). This system is able to detect the autofluorescence of NADPH (reduced form of nicotinamide adenine dinucleotide phosphate) and FAD (flavin adenine dinucleotide) at a single-cell resolution and use their ratios to determine the optical redox ratio (ORR) of the cell population as a measurement of metabolic activity (*61*, *62*). This analysis revealed that the DPT population had elevated ORR, indicative of high metabolic activity (Fig. 6, A and B). Furthermore, a random forest classifier trained to integrate the 10 metabolic parameters imaged accurately identified the DPT population from CD4 and CD8 T cells shown as a confusion matrix, ROC curve, and a Uniform Manifold Approximation and Projection (UMAP) dimensionality reduction (Fig. 6, C to E). Additional metabolic analysis using a Seahorse real-time metabolic analyzer independently confirmed that DPTs have a higher adenosine triphosphatase (ATP) production rate than CD4 and CD8 T cells, with the increased ATP production primarily coming from glycolysis (Fig. 6F). In addition to metabolic differences, we also analyzed the T cell populations for evidence of “blasting.” Blasting is characterized by increased cell size and is a marker of cellular proliferation in T cells. The DPT population displayed higher frequencies of the blasting phenotype relative to CD8 T cells in both xeno-GVHD and clinical GVHD samples (fig. S9).

### DPTs represent a GVHD-specific T cell population with minimal GVL activity

**Fig. 7.**
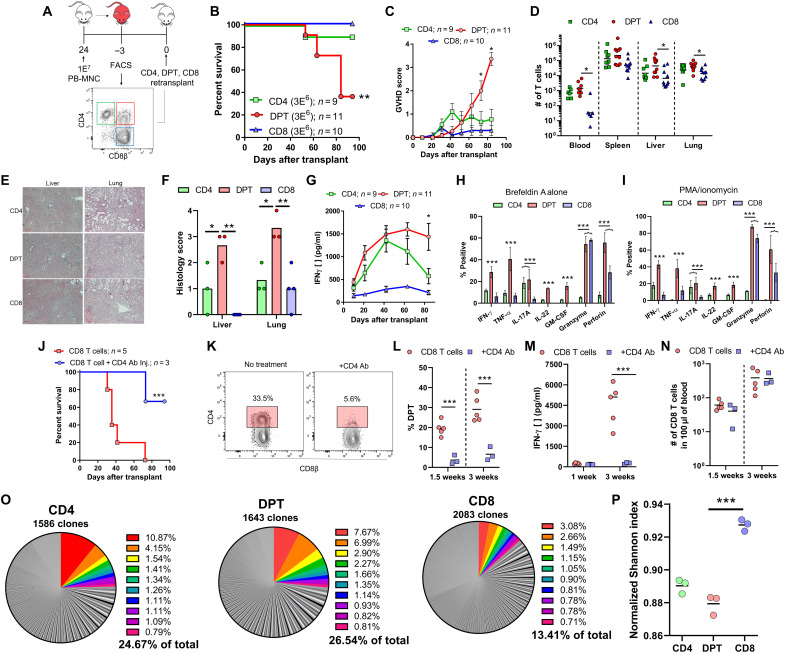
DPTs are sufficient to mediate xeno-GVHD pathology. (**A**) Schematic of experimental design. Survival curve (**B**) and GVHD score (**C**) of mice transplanted with CD4, DPT, and CD8 T cells previously isolated from five pooled xeno-GVHD mice. (**D**) Quantification of T cells in xeno-GVHD target organs at time of euthanasia. (**E** and **F**) Representative hematoxylin and eosin staining of liver and lung sections from transplanted mice (E) and histological scoring (F). (**G**) Quantification of IFN-γ levels in the plasma of transplanted mice. (**H** and **I**) Splenic T cells from 4 to 13 xeno-GVHD mice were cultured overnight with either brefeldin A alone (H) or brefeldin A with a 4-hour PMA/ionomycin stimulation (I) before intracellular cytokine staining (ICS) staining of the indicated cytokine. (**J** to **N**) Analysis of mice transplanted with human CD8 T cells and treated with a mouse anti-human CD4 antibody (Ab) (200 μg per injection, retro-orbital) at days 5 and 10 after transplant. Survival curve (J), dot plots (K), and graphical representation of the percentage of DPTs at 1.5 and 3 weeks (L). (M) Quantification of IFN-γ in the plasma and the number of CD8 T cells in the blood at 1.5 and 3 weeks (N). (**O** and **P**) TCR clonal analysis of the TRB gene segment (J) and normalized Shannon-Weiner index calculation (K). Total number of unique TRB clones is indicated, while the 10 clones with the highest frequency are colored in the pie graph. Nonparametric *t* tests were used for determining significance between T cell numbers, while parametric *t* tests were used for all other analyses. *P < 0.05, **P < 0.01, and ***P < 0.001.

To confirm the role of DPTs in the pathology of xeno-GVHD, we sorted CD4, CD8, and DPTs from xeno-GVHD mice and retransplanted each population individually into naïve mice (Fig. 7A). We observed that only the DPT population was sufficient to mediate lethal xeno-GVHD (Fig. 7, B and C). DPTs expanded rapidly after transplantation and had increased infiltration into the spleen, liver, and lungs relative to CD4 and CD8 T cells (Fig. 7, D to F). The transplanted DPT population primarily retained their DPT phenotype throughout the experiment (fig. S6). Mice transplanted with DPTs also exhibited high levels of human IFN-γ in their plasma compared to CD8 T cells (Fig. 7G). To further investigate the secretome of DPTs, we cultured human T cells from xeno-GVHD mice overnight with brefeldin A and then stimulated with or without phorbol 12-myristate 13-acetate (PMA)/ionomycin before intracellular cytokine staining. DPTs secreted a diverse repertoire of cytokines including granzyme and perforin, the inflammatory modulators interleukin-17A (IL-17A), IL-22, and granulocyte-macrophage CSF (GM-CSF), as well as the activation cytokines IFN-γ and tumor necrosis factor–α (TNF-α), indicative of a highly inflammatory T cell population (Fig. 7, H and I, and fig. S10). To further support the role of DPTs in xeno-GVHD, we transplanted isolated CD8 T cells into NSG mice followed by two in vivo injections of a mouse anti-human CD4 antibody (200 μg per injection) at days 5 and 10 after transplant. We observed that using an anti-human CD4 antibody to deplete the DPT population largely protected mice from xeno-GVHD, while all untreated mice developed lethal xeno-GVHD (Fig. 7J). The anti-human CD4 antibody treatment effectively depleted the DPT population at 1 and 3 weeks after transplant, as shown by both flow cytometry of blood samples and the measurement of IFN-γ in the plasma (Fig. 7, K to M). Antibody treatment did not alter the number of nonreactive CD8 T cells in the mouse (Fig. 7N). The one mouse from the CD4 antibody treatment group that died at 9 weeks after transplant had elevated levels of DPTs and IFN-γ starting at 6 weeks, suggesting that this mouse had only a partial depletion of the DPT population.

Clonal TCR analysis of the TRAB gene within the CD4, CD8, and DPT populations also provided evidence that the CD4 and DPT populations are experiencing clonal expansion. Both the CD4 and DPT populations had lower Shannon diversity indices and had the 10 most abundant clones representing ~25% of the entire population (Fig. 7, O and P, and table S3). While analysis of the TRAB locus supports the hypothesis that both CD4 and DPTs are experiencing clonal expansion and are being activated by a xenogeneic antigen, only the inflammatory DPT population was able to mediate lethal xeno-GVHD pathology.

**Fig. 8.**
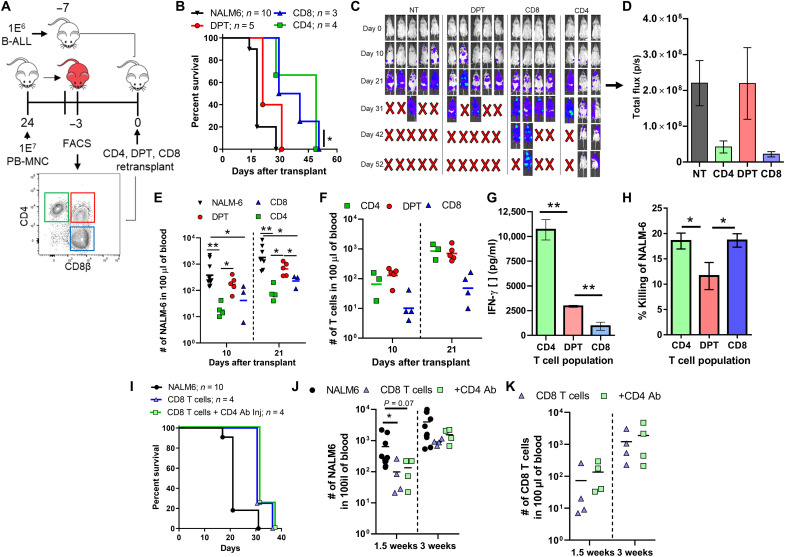
DPTs have limited GVL activity compared to CD4 and CD8 T cells. (**A**) Schematic of experiment design. The B-ALL cell line NALM-6 was transplanted into mice 7 days before human T cells. DPTs were sorted from xeno-GVHD mice, while fresh CD4 and CD8 T cells were isolated from the same donor before transplantation. Survival curve (**B**), IVIS images (**C**), IVIS quantification (**D**), number of NALM-6 (**E**), and T cells (**F**) in the blood. IVIS quantification is for day 21, while NALM-6 and T cell numbers at days 10 and 21 are shown. (**G**) Plasma IFN-γ levels were also evaluated at 3 weeks after transplant. (**H**) NALM-6 cells preloaded with calcein dye were cocultured with CD4, DPT, and CD8 T cells for 4 hours, and supernatants were quantified for the presence of calcein. Data are expressed relative to a max kill condition using Triton X-100. (**I** to **K**) Human CD8 T cells were transplanted followed by two retro-orbital injections of a mouse anti-human CD4 antibody (200 μg per dose) at days 5 and 10 after transplant. Survival curve (I), number of NALM-6 cells in the blood of mice (J), and number of CD8 T cells in the blood are shown (K). Nonparametric *t* tests were used to determine significance for the quantification of B-ALL and T cell numbers, while parametric *t* tests were used for all other analyses. *P < 0.05 and **P < 0.01.

While we have shown that DPTs are sufficient to mediate xeno-GVHD pathology, it is unclear whether they can also have activity against human blood cancers. To investigate this question, we transplanted the human B-ALL cell line, NALM-6, into mice 7 days before the transplantation of DPTs (sorted from xeno-GVHD mice) or freshly isolated human CD4 and CD8 T cells taken from the same donor (Fig. 8A). The DPT population provided no survival advantage to the mice relative to mice not receiving any human T cells, while both the CD4 and CD8 T cell populations were able to delay death (Fig. 8B). While all mice eventually succumbed to the cancer, mice receiving either CD4 or CD8 T cells had a lower cancer burden at 3 weeks after transplant when compared to DPT or no treatment controls (Fig. 8, C to E). Failure of the DPT to provide protection was not the result of failed expansion or activation, as mice receiving DPTs had equivalent numbers of T cells as mice transplanted with CD4 or CD8 T cells and the presence of IFN-γ in their plasma (Fig. 8, F and G). DPTs also exhibited a lower capacity for direct NALM-6 killing in an in vitro coculture relative to CD4 and CD8 T cells (Fig. 8H). To determine whether this difference was due to DPTs having been sorted from a xeno-GVHD mouse, we repeated this experiment using CD4 and CD8 T cells sorted from xeno-GVHD mice. We observed the same phenotype, with DPTs failing to provide a survival advantage when challenged with either the human B-ALL cell line NALM-6 or RS4;11 (fig. S11). Last, to determine whether DPTs lacked GVL activity because they developed independent of human B-ALL cancer antigens, we repeated this experiment with isolated CD8 T cells and depleted the DPT population using a mouse anti-human CD4 antibody. If DPTs also developed against human B-ALL, we should see a loss of protection when DPTs are depleted. As before, we did not observe any difference in GVL activity when the DPTs were depleted in regard to survival or the quantity of human B-ALL in the blood of mice (Fig. 8, I and J). Overall, these results suggest that this inflammatory DPT population, which develops from the CD8 T cell population after transplant as a result of antigen/xenogeneic stimulation by the TCR, may represent a T cell population that is biased toward causing GVHD pathology and has limited GVL activity.

## DISCUSSION

In this study, we identified five T cell–specific predictive biomarkers of allo-HCT outcome from a 35-patient observational study, which included a unique CD4/CD8 DPT population as a predictor of GVHD. We further investigated this DPT population in a xenogeneic transplant model system and characterized the population as being transcriptionally, metabolically, and functionally distinct from CD4 and CD8 single-positive T cells. We also provide evidence that DPTs represent chronically activated CD8 T cells that gain the effector functions of the CD4 lineage. The highly inflammatory DPT population also showed sufficiency in mediating xeno-GVHD lethality but provided no observable survival advantage to mice challenged with human B-ALL malignancies. Thus, the human DPT population represents a highly activated and pathogenic T cell population directly implicated in the pathogenesis of GVHD but not GVL activity.

We are not the first group to identify the association of DPTs with xeno-GVHD. Alhaj Hussen *et al.* also observed the development of DPTs during xeno-GVHD and that DPTs arise from the CD8 T cell population. While our study supports their findings that DPTs originate from the CD8 T cell population, their functional analysis led them to conclude that DPTs obtain a regulatory phenotype characterized by high levels of IL-10 and IL-13, low levels of IFN-γ, and high PD-1 expression (*46*). Alternatively, our analysis of the DPT population suggests that they are highly inflammatory, secreting IFN-γ, TNF-α, GM-CSF, IL-17A, IL-22, granzyme, and perforin, and are metabolically activated (Figs. 6 and 7). Alhaj Hussen *et al.* (*46*) also found no association of DPTs with allo-HCT recipients with active GVHD when blood, duodenal, or rectal biopsies were analyzed. In our observational clinical study that analyzed blood samples from patients before GVHD diagnosis, we found that DPTs were strongly linked with future GVHD diagnosis (Fig. 1).

It is not immediately clear why our two studies observed a different functional outcome for DPTs, but there are several important differences to consider. Throughout our studies, we use CD8β as a marker of the CD8 lineage, which is less promiscuous than CD8α (*39*). For example, it has been shown by others that CD4 T cells can up-regulate CD8α, but not CD8β, upon activation (*39*). With their use of γ irradiation before xenogeneic transplantation by Alhaj Hussen *et al.* (a conditioning step we have shown to not be required for xeno-GVHD), it is possible that a proportion of the DPT population they identified actually represents activated CD4 T cells. This would explain why their DPT population had high levels of IL-10 (activated T_regs_) and IL-13 (T_H_2 response) while also having IFN-γ and IL-17A expression (DPT response). In addition, in our clinical cohort, we based our analysis on the CD45RO^+^ effector/memory T cell population that allowed us to remove the additional variability of nonreactive T cells. Reanalysis of our clinical data using the CD3^+^ T cell population (similar to Alhaj Hussen *et al.*) resulted in lower resolution and weaker significance values across all of the biomarkers we tested, potentially explaining the difference between our clinical observations.

There are a variety of mechanisms that may explain the development of a human T cell population that coexpresses both CD8 and CD4, which include trogocytosis, recent thymic emigrants (RTEs), doublets, and the transient expression of CD4 on activated CD8 T cells (*58*). The use of a singlet gate during analysis and/or flow sorting as well as ImageStream visualization excludes the possibility of DPTs being an artifact of improper gating (Fig. 2C and fig. S2). We have confirmed that DPTs arise irrespective of donor and graft source, and the primary use of peripheral blood grafts in our study, which contain negligible numbers of RTEs and hematopoietic stem and progenitor cells (HSPCs), suggests that DPTs are not RTE coming from the graft. Furthermore, NSG mice lack a functional thymus, excluding the possibility that DPTs are the result of de novo hematopoiesis (*63*). Trogocytosis by CD8 T cells has been well documented but can also be excluded as DPTs arise after transplantation of isolated human CD8 T cells, wherein there is no source of human CD4 for them to acquire. The observation that CD4 is also up-regulated transcriptionally suggests that the mechanism of CD4 acquisition is molecularly driven and not acquired from the environment.

Xenogeneic transplantation is a useful model to directly investigate the biology of human immune cells, although we recognize that careful consideration must be given to xenogeneic transplantation studies. One important consideration is the nature of the antigen and antigen-presenting cells. While the specific antigen driving an allogeneic versus xenogeneic response is most likely different, the molecular response of the human T cell to the xeno-antigen can be informative on how human T cells respond to allo-antigen. As we have reviewed elsewhere, the interaction of the TCR/CD4/CD8 molecules with murine major histocompatibility complex (MHC) is fully cross-reactive (*31*). We have also shown in this study that the in vitro coculture of human CD8 T cells with murine MNCs from NSG mice can drive the development of DPTs that can also be inhibited using tacrolimus (Fig. 5, G to I). In addition, DPT development is not a direct consequence of the lymphopenic/xenogeneic environment as CD8 T cells isolated from xeno-GVHD mice do not redevelop the DPT population after retransplantation (Fig. 5, D and E). This, in addition to the decreased Shannon diversity index of the TCRs within the DPT versus CD8 populations, supports our conclusion that DPT development is driven by antigen stimulation (Fig. 7, J and K).

The T cell population is well known for its plasticity, with several other T cell populations identified in mice that share a similar function as DPTs. One population, denoted as Tc17 cells, are CD8 T cells that express a variety of CD4 lineage cytokines, such as IL-17A, IL-22, and GM-CSF (*45*). Furthermore, the directed depletion of this population was able to prevent lethal GVHD but was also shown to be noncytolytic and has minimal GVL activity (*45*). Two other studies describe how chronically activated CD4 T cells are able to coexpress THPOK and RUNX3, with CD4 T cells differentiating into MHC-II–restricted CD4^+^ cytotoxic effector cells (*39*, *64*). While these T cell populations may be transcriptionally and/or functionally similar to the human DPTs we have explored in our study, there remain important differences between each of these populations, which include the lack of CD4 expression in Tc17 cells and a difference in the starting population for CD4^+^ cytotoxic effector cells. One possibility is that these small but important differences are the result of subtle deviations between murine and human T cell biology, with a directed study comparing murine Tc17 cells and human DPTs possibly required to fully understand their similarities/differences (*65*).

In support of the xenogeneic experiments in this investigation, our 35-patient observational study of allo-HCT recipients also linked the presence of DPTs with GVHD diagnosis. The 35 patients in our cohort all received posttransplant cyclophosphamide-based GVHD prophylaxis, which is standard at our center. It is now unclear whether DPTs would also be predictive of GVHD development in allo-HCT patients receiving standard tacrolimus- and methotrexate-based GVHD prophylaxis. While patients were included in our cohort irrespective of HLA matching and conditioning regimen, additional studies will be required to validate DPTs as a predictive biomarker of GVHD in patients not treated with posttransplant cyclophosphamide (table S1).

To identify the five T cell predictive biomarkers in this study, we gated exclusively on the CD45RO^+^ T cell population (fig. S2). The gating on CD45RO^+^ T cells is based on the knowledge that CD45RO is only expressed on effector and memory T cells and the hypothesis that gating on this effector/memory T cell population would provide additional sensitivity and specificity to the potential biomarkers. With the patient’s conditioning regimen eliminating or reducing their own T cell numbers to very low levels, the CD45RO^+^ T cell population most likely represents donor T cells from the graft. Thus, we are using CD45RO as a marker to identify donor effector T cells that are activated against either host or leukemia antigen. While we acknowledge that this gating strategy is not specific for effector T cells, it does allow us to remove naïve, nonreactive T cells from our analysis, which improved the sensitivity of our biomarkers when compared to analysis of the entire T cell population.

Overall, this study supports the hypothesis that human DPTs are a highly inflammatory and pathogenic T cell population that are sufficient to mediate xeno-GVHD and strongly predictive of GVHD development in primary allo-HCT recipients. Metabolic, transcriptomic, and functional studies all identified DPTs as a T cell population with distinct inflammatory properties relative to single-positive CD4 and CD8 T cells. This highly inflammatory state resulted in lethal xeno-GVHD pathology when isolated DPTs were transplanted into naïve mice, while CD4 and CD8 T cell populations, also isolated from xeno-GVHD mice, failed to cause lethal pathology after retransplantation. This inflammatory DPT population could not control two human B-ALL cell lines though, suggesting that they have limited GVL activity. Furthermore, the increased clonality observed in DPTs compared to CD8 T cells and the capacity of tacrolimus to inhibit DPT development in vitro suggest that development of DPTs is the result of antigenic stimulation and not a direct result of a lymphopenic/xenogeneic host environment. Future studies will need to further explore the association of DPTs with other human chronic inflammatory disorders where they have already been identified, including rheumatoid arthritis, hepatitis B and C, β-thalassemia, renal carcinomas, breast cancers, and islet graft rejection (*47*–*53*). In conclusion, DPTs represent a unique T cell population whose presence should be directly investigated in other human chronic inflammatory diseases.

## MATERIALS AND METHODS

### Study design

The objective of this study was to identify T cell–based predictive biomarkers of GVHD and relapse following allo-HCT and to describe the origin and function of human CD4^+^/CD8^+^ DPTs. To achieve these aims, we performed an observational study of 35 allo-HCT recipients and performed several in vivo and in vitro experiments using a xenogeneic transplantation model system. We tested the hypothesis that DPTs arise from antigenic stimulation and that they are sufficient to mediate xeno-GVHD pathology. The number of donors, mice, and replicates are indicated in the figure or figure legend. No data were excluded from this analysis. For mouse experiments, equal numbers of male and female mice were used in each experiment, and specific experimental conditions were not grouped by cage to facilitate appropriate randomization.

### Isolation of primary human cells

Human peripheral blood was collected from healthy consenting donors according to Institutional Review Board (IRB) protocol 2014-0806 (J.E.G.) and 2017-1070 (C.M.C.). Leftover and deidentified remnants of human bone marrow and G-CSF mobilized peripheral blood grafts used for allo-HCT procedures at our clinic were collected under IRB protocol 2016-0298 (P.H.). Deidentified human umbilical cord blood was collected from the Medical College of Wisconsin’s cord blood bank. All human blood samples were diluted 1:1 with leukocyte isolation buffer [phosphate-buffered saline (PBS), 2 mM EDTA, and 2% fetal bovine serum (FBS)] before Ficoll density gradient centrifugation (1100*g* for 15 min with zero brake). When indicated, STEMCELL Technologies RosetteSep T cell, CD4, or CD8 enrichment kits were used.

### Clinical sample collection

All patients receiving an allo-HCT at the University of Wisconsin-Madison were prospectively enrolled between October 2020 and August 2021 on the minimal-risk IRB protocol 2020-1490 (C.M.C.). A total of 35 patients were enrolled in this study. Eligible patients had to receive posttransplant cyclophosphamide for GVHD prophylaxis at day +3/+4. Conditioning regimen, HLA matching status, graft source, and patient disease type were all nonexclusion criteria for this study. Patients were divided into four respective categories based on their first reportable allo-HCT outcome: relapse of primary disease, no GVHD, grade 1 GVHD, or grade 2 to 4 GVHD. Patient assignment into each respective GVHD category is based on their highest GVHD grade achieved between days 0 and 100 after transplant, while a relapse event must have occurred within 1 year of transplant. In our cohort, one patient developed grade 2 GVHD at day 78, was treated with steroids, and later relapsed at day 176. Since it is unknown how the treatment of steroids influenced this patient’s rate of relapse, this patient was placed in the grade 2 to 4 GVHD group.

Excess blood from study patients was set aside after initial use and stored at 4°C for 3 to 10 days before processing and was never frozen. A total of 0.5 ml of blood was aliquoted from each patient’s collection tube for each flow cytometry test and centrifuged at 600*g* for 4 min at 4°C. The cell pellet was resuspended in 1.5 ml of 1× red blood cell (RBC) lysis buffer (BioLegend), vortexed, and rested for 2 min before washing with 1 ml of PBS and centrifugation. Supernatant was disposed of, and a second round of RBC lysis was performed. Afterward, the cell pellet was resuspended in 1.5 ml of flow buffer (1× PBS + 2% FBS) and aliquoted into three flow cytometry tubes for staining.

### Transplantation of human cells into NSG mice

All human cells were washed and resuspended in 150 μl per mouse of sterile 1× PBS before retro-orbital injection. Equal numbers of male and female immunodeficient NSG (NOD.Cg-*Prkdc^scid^ Il2rg^tm1Wjl^*/SzJ, The Jackson Laboratory) between 8 and 16 weeks of age were used for all experiments. All mice were weighed and monitored weekly for visible signs of GVHD with a scoring system carried out as follows: 0 = no signs of GVHD; 1 = 2 to 5% weight loss; 2 = 6 to 9% weight loss; 3 = 10 to 14% weight loss; 4 = ≥15% weight loss. Blood was regularly drawn at 1, 3, 6, 9, and 12 weeks after transplant by retro-orbital bleeding. At the time of euthanasia, the spleen, one lobe of the liver, lungs, and femur were regularly collected for further processing.

### Flow cytometry and cell sorting

All cells were stained in flow buffer (PBS and 10% FBS) before quantification on the Thermo Fisher Scientific Attune NxT Flow Cytometer and analysis on FlowJo V10. Antibody clones include mCD45 (A20), ICOS (C398.4A), T-bet (4B10), LAG3 (11C3C65), FASL (NOK-1), CD4 (OKT4), CD69 (FN50), OX40 (Ber-ACT35), CD27 (O323), pSTAT4 (p38), RORγt (Q21-559), CD44 (BJ18), NKG2D (1D11), PD-1 (EH12.2H7), THPOK (ZFP-67), TIGIT (A15153G), CD45RO (UCHL1), TIM3 (F38-2E2), pSTAT6 (A15137E), IL-22 (2G12A41), GATA3 (16E10A23), CD8α (RPA-T8), CD8β (SIDI8BEE), CD25 (BC96), 4-1BB (4B4-1), CD28 (CD28.2), RUNX3 (SD0803), CTLA4 (BNI3), PanHLA (w6/32), and CD3 (UCHT1). Visualization of cells was completed using ImageStream Mark II.

For cell sorting, cells were collected from the blood, spleen, liver, and lungs of mice transplanted with a lethal dose of PB-MNC approximately 4 weeks prior. Processed tissues were Ficolled as described above to obtain mononuclear cells. Human cells were isolated from the combined mononuclear cells using a STEMCELL Technologies EasySep Mouse/Human Chimera isolation kit. Human cells were then stained in sterile PBS with the human CD4 (OKT4) and CD8β (SIDI8BEE) antibodies before sorting on a BD FACSAria cell sorter. Cells were then maintained in cell culture for 2 to 3 days before transplantation and/or expansion.

### Cell lines and cell culture

The human B-ALL line RS4;11 and NALM-6 were purchased from the American Type Culture Collection and cultured using standard RPMI-10 media (RPMI base media, 10% FBS, 1× penicillin-streptomycin, 1 mM non-essential amino acids (NEAA), and 1× GlutaMAX). After flow sorting, isolated human CD4, CD8, and DPT populations were expanded by coculturing with a 10× dose of γ-irradiated (25 Gy) human mononuclear cells from a third-party donor with phytohemagglutinin (5 μg/ml) in RPMI-10 media supplemented with human IL-2 and IL-7 (5 ng/ml). RPMI-10 media supplemented with IL-2 and IL-7 was added/changed approximately every 3 to 4 days. Human CD8 T cells were cocultured with mouse MNC isolated from the spleen, liver, and lungs of NSG mice after Ficoll separation. Cells were cultured with IL-2 and IL-7, as previously highlighted. Tacrolimus was added at a concentration of 20 μM (MedChemExpress).

### RNA sequencing

T cells from five GVHD mice were pooled before flow sorting of CD4, CD8, and DPTs. Pooled T cell populations were then split into three replicates for RNA isolation. All RNA isolation and sequencing was performed by the Gene Expression Center at the University of Wisconsin-Madison. RNA was isolated with Qiagen RNeasy kit before a quality control check using an Agilent Bioanalyzer and NanoDrop One spectrophotometer. mRNA libraries were prepared using the TruSeq stranded mRNA kit and sequenced on an Illumina NovaSeq 6000 platform to 30 million reads per sample. Sample reads were processed by the University of Wisconsin Biotechnology Center.

### Enzyme-linked immunosorbent assays

Mouse blood samples were diluted with 50 μl of 1× PBS before centrifugation with 60 μl of the diluted plasma stored at −20°C. Plasma was further diluted 1:5 before enzyme-linked immunosorbent assay (ELISA) with assay buffer (PBS, 1 mM Tween 20, and 5% FBS). IFN-γ ELISAs used the MD-1 clone for coating and a biotinylated 4S.B3 clone for detection before visualization with a streptavidin–horseradish peroxidase antibody (BioLegend).

### Western blot

An equivalent number of T cells (1 × 10^6^ cells per group) were lysed in Laemmli sample buffer with β-mercaptoethanol (Bio-Rad, CA). Total cell lysates for each sample were resolved on bis-tris 4 to 12% gel (Thermo Fisher Scientific, NuPAGE) and transferred to polyvinylidene fluoride membranes (Millipore, Billerica, MA). The membranes were blocked in LI-COR blocking buffer (LI-COR, NE). Immunoblotting was performed by incubating the membranes with anti-human RUNX3 (mouse, Cell Signaling Technology, MA), anti-human ThPOK (Rabbit, Cell Signaling Technology, MA), and anti-human glyceraldehyde-3-phosphate dehydrogenase (GAPDH) (rabbit, Cell Signaling Technology, MA), according to the manufacturer’s recommendations. The membranes were then washed with tris-buffered saline with tween-20 (TBST) and incubated with fluorescent secondary antibodies (LI-COR, NE), and the immunoreactive bands were visualized using the Odyssey CLx imaging system (LI-COR, NE).

### Two-photon lifetime imaging of NADPH and FAD

A custom-built inverted multiphoton microscope (Bruker Fluorescence Microscopy, Middleton, WI, USA) was used to acquire fluorescence intensity and lifetime images. The equipment consists of an ultrafast laser (Spectra Physics, Insight DSDual), an inverted microscope (Nikon, Eclipse Ti), and a 40× water immersion (1.15 numerical aperture, Nikon) objective. NADPH and FAD images were obtained for the same field of view. Using the masks, single-cell features were extracted including biexponential fit parameters (τ_1_, τ_2_, α_1_, and α_2_) for NADPH and FAD, NADPH τ_m_, FAD τ_m_, and the ORR. Features were calculated on a per-cell level by averaging all the pixels across each cell. A minimum of 100 cells were analyzed per condition to obtain statistically robust results. Additional information can be found in the Supplementary Materials.

### Seahorse metabolic assay

ATP production rate was calculated from flow-sorted CD4, CD8, and DPT using an Agilent Seahorse XF real-time ATP rate assay kit using a Seahorse XF/XFe96 analyzer and following the manufacturer’s instructions.

### Bioluminescent imaging

A total of 1 × 10^6^ luciferase-positive NALM-6 cells were injected retro-orbitally 7 days before human T cell transplantation, as described above. Luminescence detection was gathered at the indicated times using an IVIS Spectrum in vivo imaging system (PerkinElmer). Briefly, mice were anesthetized and intraperitoneally injected with 100 μl of d-luciferin at 30 mg/ml, with luminescence obtained approximately 15 min later.

### Statistics

Graphs and statistical tests were completed using GraphPad Prism 6. All significance tests were performed using either unpaired parametric (for linear data) or nonparametric (for logarithmic data) *t* tests. For the biomarker multivariate analysis, we used the multiplications of the empirical cumulative distribution factor for each variable to achieve a combined score with the R language. AUC-ROC was used to determine the optimal direction of the exdf (< or >) for each variable. Generalization performance was estimated using leave-one-out cross-validation. For the RNA-seq analysis, each fastq file is assessed for quality control issues and trimmed to remove adapter sequences that were not removed by the initial demultiplexing. After trimming and quality controlled, the reads were aligned to the human genome using the splice-junction aware read aligner STAR. The software package RSEM was used to generate normalized read counts for each gene and its potential isoforms. Genes with very low expression, which would otherwise reduce statistical power, were filtered out. The program EdgeR is then used to perform differential gene expression analysis.

### Study approval

All work involving human cells and tissues was performed in accordance with IRB protocol 2014-0806 (C.M.C.). All animal work was performed in accordance with UW-Madison Institutional Animal Care Use Committee protocol M005915 (C.M.C.).

## Notes

View/request a protocol for this paper from *Bio-protocol*.
